# Furthering the Validation of Passive Detection of Cigarette Smoking

**DOI:** 10.1093/ntr/ntac251

**Published:** 2022-10-28

**Authors:** Andy Skinner, Christopher Stone

**Affiliations:** Integrative Cancer Epidemiology Programme, University of Bristol, Bristol, UK; UK Medical Research Council Integrative Epidemiology Unit at the University of Bristol, Bristol, UK; Bristol Medical School, University of Bristol, Bristol, UK; Integrative Cancer Epidemiology Programme, University of Bristol, Bristol, UK; UK Medical Research Council Integrative Epidemiology Unit at the University of Bristol, Bristol, UK; School of Psychological Science, University of Bristol, Bristol, UK

Systems for passive detection of cigarette smoking offer a number of benefits compared with traditional methods for recording smoking behavior. They are non-intrusive, have low participant burden, and are not subject to recall and desirability biases.^1,2^ We recently developed a smartwatch-based system which uses data from the watch’s motion sensors to passively detect puffs on a cigarette, and from the pattern of puffs identifies and records an instance of smoking a cigarette (“stopWatch”).^3^

When validating the performance of these systems it is important this is conducted in free-living conditions.^4^ We initially conducted a validation of stopWatch running on an LG smartwatch among 13 participants who were asked to smoke normally as they went about their normal lives for 24 h. To capture ground truth data (the actual smoking behavior of the participant), participants completed a paper diary, and used an app on the smartwatch to confirm when a stopWatch cigarette smoking detection was correct (true positive), and to log when smoking a cigarette was missed (false negative). From this, on average 11.9 cigarettes/participant were smoked, with an overall total of 155 cigarettes smoked. We compared cigarettes smoked detected by stopWatch with the ground truth data. Mean sensitivity of the system (also referred to as recall) was 71% (95 CI = 63–78%), and the positive predictive value (PPV, also referred to as precision) was 86% (95 CI = 78–93%).

Having completed that validation, we were faced with a dilemma: what do we do when things change? What happens when the smartwatches we use become obsolete and we need to use different smartwatches? What if we want to use stopWatch in a setting different to the normal domestic settings in which people typically complete these evaluations? Can we be confident the performance of the system will remain unchanged?

In terms of changes in smartwatch, we included in the design of our system an intermediate processing stage between the motion sensor outputs and the smoking classification stage to normalize the dynamic range of the motion sensor outputs. To test this we conducted another validation, this time with the system running on a Mobvoi TicWatch C2 smartwatch.^5^ The process followed was the same as our initial validation, but because of COVID-19 restrictions participant briefings, familiarization with equipment, and debriefing were conducted remotely by video calls rather than face-to-face. From 13 participants smoking on average 12.9 cigarettes/participant and a total of 168 cigarettes the mean sensitivity was 78% (95 CI = 72–85%) and PPV was 88% (95 CI = 82–94%), so very similar to the performance of stopWatch on a LG smartwatch. This gives us confidence that a moderately simple addition to the processing pipeline can offer some resilience to changes in smartwatch platform.

Perhaps the biggest distinction in settings in most people’s daily lives is between their home and workplace. To explore the performance of stopWatch in the workplace we decided to push the limits of the system, and tested it among employees of a construction company in Southwest England.^6^ Only six participants could be recruited in the timescales, and they smoked on average 6.5 cigarettes/participant/day over 4 days, with a total of 167 cigarettes smoked. Overall, performance fell dramatically, with mean sensitivity of 31% (95 CI = 13–49%). Poor levels of ground truth data entry may have influenced these metrics, and meant PPV could not be computed. Other issues identified were not keeping the battery properly charged, and not re-starting the watch and system correctly after a flat battery. Importantly, these could be targeted by improving participant briefings and modifying the design of future systems.

In completing these validations, we made two broad observations about the process of validation itself. The first was that it is a resource intensive and time-consuming activity, and any steps that can be taken to reduce the time and resources required but maintain (or improve) the integrity of the exercise should be welcomed. One such step is to conduct the validation exercise remotely, as we did in the second validation study described here, and which appeared to work very successfully. This removes the need for face-to-face sessions and laboratory/clinic resources. It also enables recruitment from a much broader participant base, including seldom reached communities in society, which helps mitigate issues around digital exclusion.

The second was that perhaps the most challenging aspect of the validation process is establishing the ground truth used to determine system performance. For measures such as physical activity, research grade wearables acknowledged as gold standards in that field can be worn for short periods to provide reliable data against which the outputs of new devices can be compared.^7^ For more complex behaviors like smoking, equivalent gold standards for measurement in free-living conditions do not exist. In these cases, there is more reliance on the participants completing diaries, or using apps on phones or watches, and so the data are less reliable. One approach that could potentially help is the use of on-body cameras. These are small camera devices worn attached to clothing or around the neck, that capture objective footage of whatever the participant is doing, in free-living conditions. This approach has been used successfully to measure detailed interactions between people,^8^ and to some extent to record cigarette smoking.^9^ One of the major limitations of these methods is that they can breach privacy, not just for the participant but for those around them, so protracted use may result in issues of this kind. However, use for limited periods of time is far more likely to be tolerated, and we suggest the method warrants further investigation in the context of validation studies.

Validating performance is key to the development, use and refinement of systems for passive detection of behaviors like smoking. It is important validations are conducted in a manner that reflects the variety of ways a system will be used in real life, and consider factors that will inevitably be encountered during the lifetime of the system, including the need to update hardware, operating systems, and software. This can take considerable time and resources, and new methods for reducing the burden of validation need to be identified and embraced.

## Supplementary Material

ntac251_suppl_Supplementary_Taxonomy-form
